# Functional Biodiversity of Yeasts Isolated from Colombian Fermented and Dry Cocoa Beans

**DOI:** 10.3390/microorganisms8071086

**Published:** 2020-07-21

**Authors:** Johannes Delgado-Ospina, Samantha Triboletti, Valentina Alessandria, Annalisa Serio, Manuel Sergi, Antonello Paparella, Kalliopi Rantsiou, Clemencia Chaves-López

**Affiliations:** 1 Faculty of Bioscience and Technology for Food, Agriculture and Environment, University of Teramo, Via R. Balzarini 1, 64100 Teramo, Italy; jdelgadoospina@unite.it (J.D.-O.); samantha.triboletti@studenti.unite.it (S.T.); aserio@unite.it (A.S.); msergi@unite.it (M.S.); apaparella@unite.it (A.P.); 2 Grupo de Investigación Biotecnología, Facultad de Ingeniería, Universidad de San Buenaventura Cali, Carrera 122 # 6-65, 76001 Cali, Colombia; 3 Department of Agricultural, Forestry and Food Sciences, University of Turin, Largo Paolo Braccini 2, 10095 Torino, Italy; valentina.alessandria@unito.it

**Keywords:** Cocoa microbiota, *Saccharomyces cerevisiae*, *Pichia kudriavzevii*, Polyphenols, Trehalose, thermotolerance, enzyme activities

## Abstract

Yeasts play an important role in the cocoa fermentation process. Although the most relevant function is the degradation of sugars and the production of ethanol, there is little understanding of the enzyme activities and attributes that allow them to survive even after drying. The present study explored the functional biodiversity of yeasts associated with *Criollo* Colombian cocoa fermented beans, able to survive after drying. Twelve species belonging to 10 genera of osmo-, acid-, thermo-, and desiccation-tolerant yeasts were isolated and identified from fermented and dry cocoa beans, with *Pichia kudriavzevii* and *Saccharomyces cerevisiae* standing out as the most frequent. For the first time, we reported the presence of *Zygosaccharomyces bisporus* in cocoa fermented beans. It was found that resistance to desiccation is related to the different degradation capacities of fermentation substrates, which suggests that associative relationships may exist between the different yeast species and their degradation products. Besides, the increased thermotolerance of some species was related to the presence of polyphenols in the medium, which might play a fundamental role in shaping the microbial community composition.

## 1. Introduction

Cocoa is one of the most important agricultural export commodities throughout the world, which forms the backbone of the economies of some countries in Latin America and Africa [1]. The International Cocoa Organization (ICCO) estimated that more than 4.0 thousand tonnes of cocoa beans were produced worldwide in 2017/2018 [2]. Starting from cocoa beans, cocoa production includes a series of processes such as fermentation, drying, roasting, dutching, conching, and tempering. Although the quality of cocoa powder is the result of several factors, it is well recognized that fermentation and drying are fundamental for the flavour of the product. In fact, during fermentation bacteria and yeasts act in a very well defined and complex microbial succession, in which the performance of the different species drives the degradation of the pulp bean that contributes as a precursor of chocolate flavour [3]. In particular, yeasts are the first microorganisms that grow at the beginning of fermentation and dominate for the first 30–40 h [4]. They are considered as fundamental for the cocoa bean fermentation, playing different important roles such as: the breakdown of citric acid present in the pulp, the secretion of pectinases that cause the pulp degradation, the production of ethanol through sugars fermentation (mainly glucose) under low-oxygen conditions, which kills cocoa bean cotyledons, the production of different organic acids by different metabolic pathways, and the production of important VOCs that diffuse into the interior of the beans and react with the substances responsible for the flavour of the final product during the subsequent roasting process [5]. During fermentation, the temperature increases up to 48–52 °C between 57 and 80 h, and only marginal yeasts growth is observed. Following fermentation, cocoa beans are dried in the sun or in mechanical dryers to about 5% moisture in the cotyledons and 7% moisture in the shell [6].

Different authors have studied the biodiversity of the bacteria and yeasts involved in cocoa fermentation. In particular, studies on yeast diversity revealed the presence of several species, among which the most frequently reported belonged the genus C*andida*, *Kluyveromyces*, *Hansenula*, *Hanseniaspora*, *Kazachstania, Meyerozyma*, *Pichia*, *Rhodotorula*, *Saccharomyces*, *Saccharomycopsis*, *Schizosaccharomyces*, *Wickerhamomyces*, and *Cryptococcus* spp. [3,7,8,9]. Nevertheless, very few authors explored the biodiversity after drying. Hamdouche et al., [10] reported that most of the yeasts species that are present during fermentation disappeared during the drying process, and only few species such as *Hanseniaspora opuntiae*, *Candida insectorum, Pichia kudriavzevii, Pichia sporocuriosa,* and *Issatchenkia hanoiensis* can survive during drying.

As great part of the farmers performs drying under environmental conditions, cocoa beans are largely influenced by environmental factors, especially in the summer. There is a production break during July and August, due to a decline in the productivity and quality of cocoa bean. Therefore, the objective of this experimental study was to explore the phenotypic biodiversity of yeasts microbiota associated with *Criollo* Colombian cocoa fermented beans, able to survive to the drying process. These findings should be immensely helpful to understand yeasts functions during spontaneous fermentation, considering that phenotypic diversity in cocoa yeasts populations could be exploited industrially [11].

## 2. Materials and Methods

### 2.1. Raw Materials

Nineteen samples of fermented and dried cocoa beans (*Theobroma cacao* L.) of *Criollo* variety, coming from smallholder farms in the South Western region of Colombia, were used as raw materials. About 2.0 kg of each sample were collected during the first annual production cycle in the months of April and May, at the beginning of the rainy season. The samples were collected after fermentation and sun drying, carried out according to local practices (Table S1). All cocoa beans samples were randomly selected from different sites in the storage, packed in sterile polypropylene bags, then refrigerated at 4–6 °C and analysed within 24 h from sampling. In cocoa beans, the pH was measured before and after drying, placing the beans in distilled water in a ratio 1:1 with an electrode probe connected to a pHmeter (model 507 Crison, Barcelona, Spain). Water activity (a_w_) was measured at 25 °C placing the beans inside the sample chamber, using an electrolytic hygrometer (Hygropalm, Rotronic, Bassersdorf, Switzerland), and moisture was determined in an oven at 105 °C (constant weight).

### 2.2. Yeasts Enumeration and Isolation

Yeasts counts were determined in duplicate using 10.0 g of fermented cocoa beans suspended in 90.0 mL of Phosphate Buffered Saline solution (PBS). Samples were homogenized and successively appropriate dilutions were made up. Then, 0.1 mL aliquot of each dilution were plated into YPD Agar (yeast extract 10.0 g·L^−1^, peptone 20.0 g·L^−1^, dextrose 20.0 g·L^−1^ and agar 15 g·L^−1^) and DG-18 medium (Dichloran Glycerol Agar) (Casein Enzymatic Digest 5.0 g·L^−1^, D-Glucose 10.0 g·L^−1^, Monopotassium Phosphate 1.0 g·L^−1^, Magnesium Sulfate 0.5 g·L^−1^, Dichloran 0.002 g·L^−1^, Glycerol 220.0 g·L^−1^, Agar 15.0 g·L^−1^, final pH 5.6 ± 0.2 at 25 °C). Both media were added with chloramphenicol (100 μg·L^−1^). Plates were incubated at 30 °C for 2 days. Either 50% of the colonies were selected or, if the plate contained less than 10 colonies, all of them were selected, according to Harrigan and McCance [12]. Colonies of different morphologies were randomly recovered, purified, and sub-cultured onto YPD Agar for subsequent identification. The purified isolates were preserved at −80 °C in 20% *V*/*V* glycerol/YPD broth.

### 2.3. Molecular Identification

DNA was extracted by using the *Yeast DNA Extraction Kit* (Thermo Scientific^TM^, 78870). The DNA of all isolates was subjected to restriction fragment length polymorphism (RFLP) of the region ITS1-5.8S rRNA-ITS2 (ITS) [13]. The ITS region was amplified with primers ITS1 (5′-TCCGTAGGTGAACCTGCGG-3′) and ITS4 (5′-TCCTCCGCTTATTGATATGC-3′) [14]. The reaction mix was of 50 µL and contained 10 mM Tris-HCl (pH 8.3), 50 mM KCl, 1.5 mM MgCl_2_, 0.2 mM of deoxynucleoside triphosphates (dNTPs), 1.25 U of Taq Polymerase (Applied Biosystems, Milan Italy), 0.2 µM of each primer and 100 ng of template DNA. Amplification was carried out using a PTC-200 DNA Engine MJ Research thermal cycler (Biorad, Milan, Italy), as described by [13], and the PCR products were checked by electrophoresis on 1.5% (*w*/*v*) agarose gel. The PCR products were subsequently digested by endonucleases *Hinf*I, *Hae*III, *Cfo*I (Promega, Milan, Italy), according to the supplier’s instructions. The restriction fragments were separated by electrophoresis in 3% agarose gel and stained with ethidium bromide. PCR and RFLP fragment lengths were used for identification of yeasts by comparing the restriction bands with those available in literature [13,15,16,17]. Identification to the species level was confirmed by sequencing the D1-D2 loop of the 26S rRNA encoding gene, after amplification using primers NL1/NL4 [18] to obtain a polymerase chain reaction (PCR) product, which was sequenced by a commercial facility (Eurofins, Hamburg, Germany).

Yeast diversity in each sample was measured using the Shannon (Ho) index = −Σ (*n*_i_/*n* × ln (*n*_i_/*n*), where *n*_i_ is the number of individuals of the taxon i and *n* is the total number of individuals [19].

### 2.4. Screening of the Yeast Strains for the Enzyme Activities

Purified yeast strains were inoculated in the YPD medium (yeast extract 10.0 g·L^−1^, peptone 20.0 g·L^−1^, and dextrose 20.0 g·L^−1^) and incubated at 28 °C for 48 h. The cells were centrifuged at 6000 *g* for 10 min at 4 °C. The pellet was re-suspended in 1.0 mL of physiological water solution and standardized to reach a final load of about 10^5^ CFU·mL^−1^, by spectrophotometric measurements at 620 nm (Jenway 6305, Stone, UK) and confirmation of the load by means of plate counts on YPD agar incubated at 28 °C for 24 h.

The evaluation of the different enzyme activities was carried out inoculating 20 µL of the standardized cells in the plates containing the particular substrates able to detect the enzyme activities reported below.

Lipolytic activity was evaluated according to the method reported by [20] by substituting pork fat with cocoa butter. β-glucosidase activity was detected by growing the test yeast strains on Agar containing arbutin as the sole carbon source as reported by [21]; a black colour was produced in the medium by the colonies producing β-glucosidase. Xylanolytic and laccase activities were performed following the method proposed by [22], where xylan degradation appeared as a yellow-opaque area around the colonies. Chitinase, pectinase, and cellulase activities were carried out following the method reported by [23]. Lignin modifying enzymes, evidenced by decolourisation of Remazol Brilliant Blue R (RBBR), were positively correlated with production of the polyphenol oxidases lignin peroxidase, Mn-dependent peroxidase and laccase, as described by [24].

### 2.5. Growth at 37 °C and 46 °C in Cocoa Pulp Simulation Medium

The yeasts biomass from the 24 h cultures grown in YPD was separated from the medium by centrifugation at 5000 rpm, and subjected to three washing steps under sterile conditions, and successively the cells were suspended in liquid medium to reach OD_600_ 0.5. The yeasts suspension were inoculated into three mediums and were grown at 30 °C, 37 °C and 46 °C for 8 days. The first medium was made of plates containing cocoa pulp simulation medium (CPSM) (pectin 10 g·L^−1^, (NH_4_)_2_SO_4_ 2.8 10 g·L^−1^, KH_2_PO_4_ 1 g·L^−1^, MgSO_4_. 7H_2_O 0.5 g·L^−1^, FeSO_4_. 7H_2_O 0.01 g·L^−1^, K_2_HPO_4_ 6.0 g·L^−1^, glucose 20.0 g·L^−1^, fructose 30.0 g·L^−1^, saccharose 6.7 g·L^−1^, lactic acid 1.0 g·L^−1^, acetic acid 1.4 g·L^−1^, citric acid 1.0 g·L^−1^, yeast nitrogen base 2.0 g·L^−1^, ethanol 2.0 g·L^−1^, with pH adjusted to 5.8); the second medium was made of plates containing CPSM with polyphenols (mix of cocoa polyphenols 0.051% containing catechin 1136 mg·L^−1^, epicatechin 1441 mg·L^−1^, and isoquercetin 539 mg·L^−1^); the third medium (Control) was made of plates containing YPD medium.

In addition, we were interested to know whether the thermotolerant yeasts varied in the capability to accumulate trehalose after a mild heat stress of 46 °C, miming temperatures that could be present during the drying of cocoa fermented beans. To this purpose, a representative number of strains for each species was subjected to the analysis. Thus, 100 mL flasks with a final volume of 50 mL of CPSM were inoculated at OD_600_ 0.1 with the cells obtained from a 24 h pre-culture grown in YPD. The cell suspensions were exposed to 46 °C and examined after 0 and 2 h of incubation. After the incubation time, an aliquot of 5 mL was sampled and the yeast biomass was separated from the medium by centrifugation at 5000 rpm × 10 min, then subjected to three washing steps (cells were resuspended in sterile physiological solution and then centrifuged) under sterile conditions.

Intracellular trehalose content was measured using a Trehalose Assay Kit (Megazyme, Wicklow, Irland) after the rupture of the cells with liquid nitrogen, following the instructions given by the producers.

### 2.6. Statistical Analysis

The analysis of variance (ANOVA) and the Tukey method for mean separation, with a confidence level of 95% (α = 0.05), were used to evaluate differences among the samples (Statistica 12.0 program, 2013). Heatmap was performed using ClustVis web server [25].

## 3. Results and Discussion

### 3.1. Yeast Counts and Water Activity

Measurements of pH values, *a_w_*, moisture and viable yeast counts (that were similar on plates of either YPD or DG-18 agar), were performed in samples of cocoa beans, fermented and sun dried in different localities of South West Colombia. Drying was performed as usual in naturally fermented cocoa, and that is in uncontrolled temperature and humidity conditions in which the moisture of cocoa beans diminishes thanks to direct solar radiation and natural air circulation.

As shown in Table 1, significant differences were observed in yeast counts, *a_w_* and moisture values of the beans before and after natural drying. In fact, after 5 days, average yeast viable counts decreased from 6.59 ± 0.46 to 2.69 ± 1.96 Log CFU·g^−1^. The huge variation in the yeast counts after drying could reflect the diversity of the yeasts isolated, as some of them have demonstrated high tolerance to desiccation. As regards *a_w_* values, they decreased from 0.98 to 0.52 ± 0.11. Although it is well known that this parameter contributes to the changes in the microbial community, a non-significant positive correlation (95% confidence level) was revealed between yeast counts and *a_w_* values in all samples after drying. In the same way, moisture dropped from 42.55 ± 3.24 to 3.41 ± 1.29%. On the other hand, pH was unaffected by drying, with final values similar to those measured at the beginning of the process. These values were within the range referred to for well-fermented and dry cocoa beans (between 4.9 and 5.1) [1,26].

### 3.2. Identification of the Yeast Isolates

A total of 149 yeast isolates, representative of all of the samples after drying, were grouped by the PCR-RFLP analysis of the ITS–5.8S rDNA. Then, according to the D1/D2 26S rDNA gene sequence analysis results, they were regrouped into 12 yeast species. The results of the identification for all isolates are given in Table 2.

The presence and growth of yeasts during cocoa beans fermentation can be affected by many factors, such as initial microbiota, chemical composition of the cocoa seeds, temperature fermentation, and interactions among different microorganisms [27]. On the other hand, drying selects the osmo-, acid-, thermo-, and desiccation-tolerant yeasts naturally present in cocoa beans. In natural sun drying, other variables, such as UV rays and locally high temperatures, may also have an effect on yeast survival. In this study, the strains occurring as dominant in fermented and dried cocoa seeds were found to belong to 12 species shared among 10 genera. 

As evidenced in Table 3, the most frequent and dominant species in fermented and dried cocoa beans were *Pichia kudriavzevii* and *Saccharomyces cerevisiae* that were present in 15 out of 19 samples, representing, respectively, 40% and 28% of all the isolates, followed by *Pichia manshurica* (9%), *Candida parapsilosis* (7%), and *Wickerhamomyces anomalus* (4%). The other species were less frequently isolated and their abundance was very low. The Shannon diversity values (H’) (Table S1) of the different samples analysed were all low in the samples, suggesting that only few species were able to overcome desiccation and form colonies in the culture medium used. It is important to stress that the values of H’ index were not correlated with temperature, time, or type of material in which the sun-drying process took place. These results support the hypothesis that beside the resistance to different stresses during fermentation, and also the resistance to desiccation is important for yeast survival during drying. In fact, changes in pH, temperature, sugar content, and fermentation products or metabolites can exert a selection pressure on existing natural biotypes, favouring the strains that are better adapted to this environment [28].

A recent study [29] has reported the presence of the genera *Saccharomyces, Eremothecium, Galactomyces, Frauteria, Zygosaccharomyces, Yarrowia*, and *Kazachstania* in samples from Peru, determined by MALDI TOF/TOF mass spectrometry. Moreover, the predominance of *P. kudriavzevii* and *S. cerevisiae* was reported at the end of fermentation in cocoa from Ghana [30]. In particular, both these species are considered as the best adapted and the most relevant yeast species involved in cocoa fermentation, due to their ability to adapt to changes in pH and temperature and to the ability to metabolise pulp citric acid, as well as to their low nutritional needs. For these reasons, they were reported as the dominant species in Ghanaian, Ivorian, and Brazilian cocoa beans fermentation [28], [30,31,32,33,34].

Except for *Z. bisporus*, the presence of all the species identified in this study was frequently reported in cocoa beans fermentation [31,35,36,37].

On the other hand, *S. pombe* together with *S. cerevisiae,* was identified as one of the most abundant species in heap cocoa bean fermentation in West Africa; the two species were recorded from the beginning until the end of fermentation [33]. A recent study on Malaysian cocoa fermentation has concluded that *P. kudriavzevii* and *C. quercitrusa* were the most prevalent species [38]. In Ivory Coast, some researchers evidenced a great diversity of the yeast species, varying from one local region to another; for example, *P. galeiformis, G. geotrichum* and *W. anomalus* were isolated in cocoa fermentation from the Me region [39], while *C. intermedia, C. nacodendra,* and *H. guilliermondii* were isolated in cocoa fermentation from the Agneby-Tiassa region [32]. In addition, *T. delbrueckii, C. ethanolica* and *S. pombe* were also detected in Ivorian cocoa fermentation by Visintin et al. [33], thus explaining the variability of cocoa beans quality in Ivory Coast. In the same way, *W. anomalus* was mentioned as one of the most frequently isolated yeast species, independently from the geographical area of fermentation [35]. On the contrary, to the best of our knowledge, *Z. bisporus* was never previously detected in cocoa fermented beans.

### 3.3. Extracellular Enzyme Activity Profile Differences among the Yeasts

The selective pressure of tropical environments, in terms of temperature, pH, light, and concentration of readily available nutrients, may favour yeast biodiversity and highlight a useful technological potential [40]. Therefore, in this part of the work, we investigated the different strains for some enzyme activities such as laccase, β-glucosidase, pectinase, xylanase, cellulase, and lipase, because of their potential role in fermentation and drying of cocoa beans.

**Figure 1:**
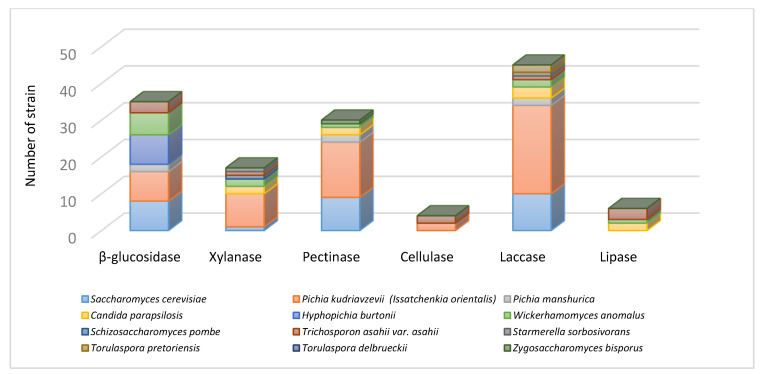
Extracellular enzyme activities showed by the different yeast species, isolated from cocoa fermented and dried beans, incubated at 25 °C for three days.

In general, intra- and inter-species differences among the yeast secretions can define the role of the different species in cocoa production. Our results evidenced marked intra and inter-species differences in the enzymes production and non-discordant results were observed in the repeated experiments. As reported in Figure 1, pectinase, laccase, and β-glucosidase were the most frequent enzyme activities detected in great part of the species, with *P. kudriavzevii* and hence *S. cerevisiae* and *W. anomalus* as the most active ones, emphasising their important role in cocoa fermentation. It is important to underline that none of the enzyme activities investigated was detected in the two *T. delbrueckii* strains tested.

Pectinases (polygalacturonase, pectinlyase, pectatelyase, and pectinesterase) are considered as the most important enzymes in cocoa fermentation. In fact, they hydrolyse the glycosidic bonds of pectin present in the cocoa seeds pulp, thus producing a fermentable carbohydrate substrate and allowing the aeration of the pulp mass and therefore enabling aerobic acetic bacteria (AAB) to grow [41]. In our study, 32 out of 144 strains showed different levels of pectinolytic activity, while only few strains belonging to *S. cerevisiae* (1/38), *P. kudriavzevi* (5/54), and *C. parapsilosis* (1/10) showed a high pectinase activity. A minimal activity was detected in *H. anomalus* and *P. manshurica*, but the enzyme was undetectable in *T. delbrueki, T. pretoriensis, S. pombe,* and *H. burtonii.* Other authors reported this activity in *S. cerevisiae* and *S. pombe* isolated from cocoa fermented beans [33,42]. Differences in pectinolytic activity were also observed [43] in *P. kluyveri* and *P. anomala* isolated from coffee fermented beans, as only one out five strains possessed a strong activity. Samagaci et al. [32] reported that 12 out of 95 strains of *P. kudriazevii* and 18 out of 18 *C. nitrativorans* strains exhibited pectin-hydrolysing enzyme activity.

Carboxymethyl cellulase and β-glucosidase are two major cellulose decomposition enzymes. In the scientific literature, yeast cellulase activity during cocoa beans fermentation was never reported before. Among our isolates (Figure 1), *S. pombe* and *P. kudriavzevii* were the only species showing this activity, which is essential for cocoa fermentation, as it contributes to developing aromatic molecules (higher alcohols, organic acids, esters, aldehydes, and ketones) [33]. Moreover, β-glucosidase enzymes produced by yeasts would contribute to degrade the cellulosic biomass of the cocoa bean pulp [44]. In our study, all the strains of the species *H. burtonii*, *T. asahii* var. *asahii*, and *W. anomalus* showed a strong β-glucosidase activity, together with *P. kudriavzevii* (8/54), *S. cerevisiae* (8/38), *P. mashurica* (2/13). On the contrary, *C. parapsilosis* did not show this activity. In other studies, also *H. uvarum* and *Metschnikowia pulcherrima* strains were found to possess β-glucosidase activity [44].

On the other hand, laccase activity is important in cocoa fermentation, as this enzyme is involved in the breakdown of lignin, hemicellulose, and cellulose present in the cocoa shell; in addition, it could also have an impact on the oxidation of phenolic substances, such as anthocyanidins present in cocoa beans [45]. This activity was the most diffused among all the tested species: *P. kudriavzevii* (24/54), *S. cerevisiae* (10/38), *P. mashurica* (2/13), W. *anomalus* (2/6), *Torulapora pretoriens* (2/2), *Z. bisporus* (1/1), *S. sorbosivorans*, and *T. asahii* var. *asahii* (1/2).

Other important enzymes are xylanases, which are able to hydrolyse complex polysaccharides into simple sugars; for this reason, the potential capability of yeasts to produce these enzymes may be favourable to cocoa bean fermentation, by reducing fermentation time. Only 17/144 strains showed this activity, with *P. kudriavzevii* as the most efficient species (9/54 positive strains), followed by S*. cerevisiae, C. parapsilosis, W. anomalus, T asahii* var. *asahii* and *S. sorbosivorans*. This is the first time in which xylanase activity has been reported in yeasts involved in cocoa fermentation.

On the other hand, by degrading cocoa beans lipids, lipases cause the increasing of free fatty acids concentration, with a negative impact on the chocolate flavour, developed during fermentation [3]. Lipase activity was not very diffused in the different tested strains, as this activity was only revealed in some strains of *C. parapsilosis, T. asahii* var. *asahii*, and *W. anomalus.*

*H. burtonii, T. pretoriensis*, and *P. manshurica* were positive to β-glucosidase, pectinolytic and laccase activities, while S*. sorbivorans* strains were positive only to xylanase and laccase activities. Concerning *C. parapsilosis,* although there are only few studies about its presence in the indigenous cocoa beans fermentation microbiota, this species is characterised by pectinolytic activity and production of organic acids and volatile aroma compounds [5].

**Figure 2:**
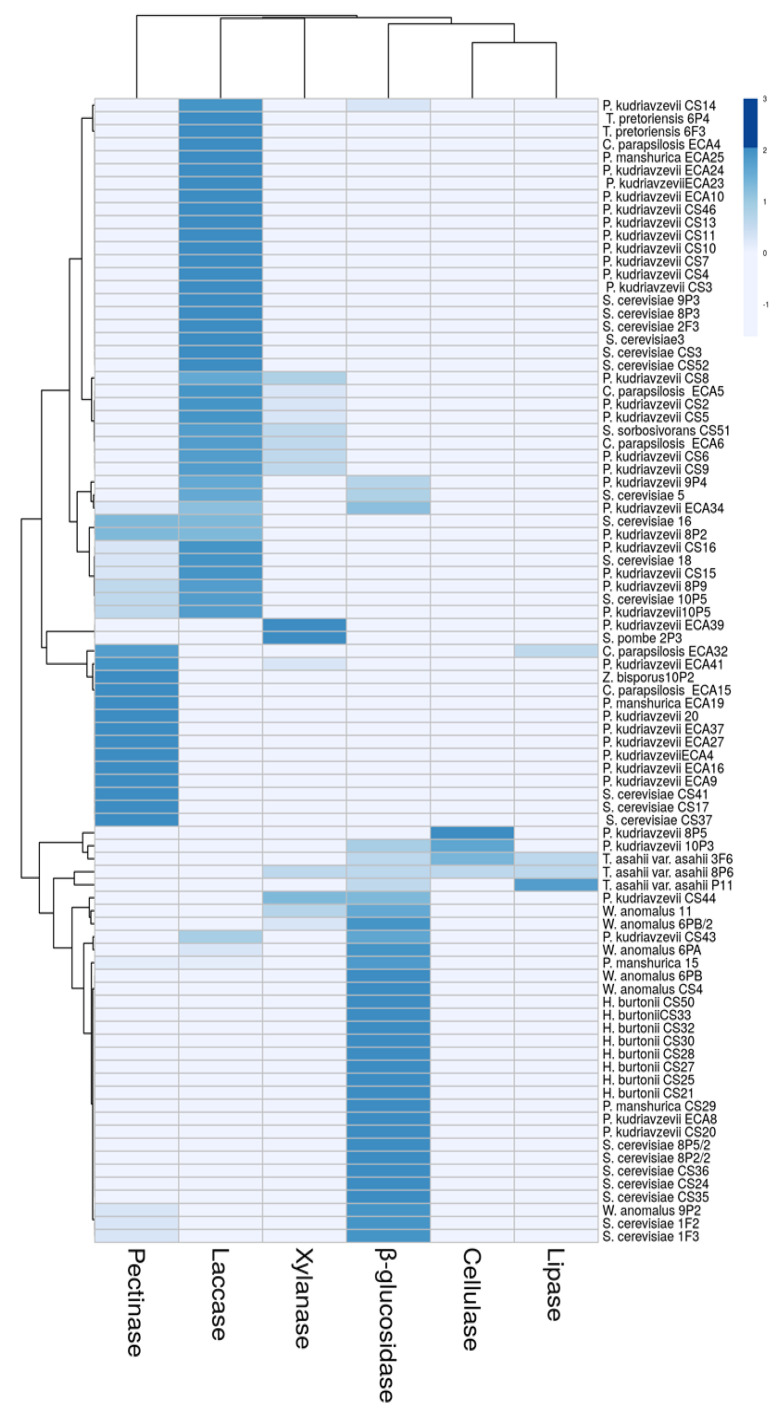
Heatmap showing the extracellular enzyme activity profiles correlation, displayed by the strains isolated from fermented and dry cocoa beans in selective solid medium incubated at 25 °C for 3 days.

The intensity of the developed colour in the plates, observed by a visual analysis, could provide additional information, as we assume that the species will differentially produce enzymes that will reveal the dissimilarities among the species. With the aim of proving this hypothesis, hierarchical clustering analysis (HCA) was performed (Figure 2). All the investigated enzyme activities were included in the analysis. We observed that sample clustering was primarily driven by the species, but the enzyme activity also played a role, creating clusters within each species.

The heatmap indicates that a reduced number of enzyme activities drives the observed clustering pattern (Figure 2). In fact, β-glucosidase, laccase and pectinase were the principal enzymes that characterised the cluster of the yeasts isolated from Colombian cocoa beans. The pattern of the enzyme activities observed in the yeasts is tied to their functional capacities. Interestingly, we detected differences in the yeast metabolism on the tested substrates as a function of unique anabolic or catabolic substrates. Nevertheless, the ability to degrade polysaccharides varied significantly within the strains. It is possible to observe that *T. asahii* var. *asahii* appeared as a distinct group, separated from the other strains that showed the highest hydrolytic activity towards cocoa butter and moderate cellulose and β-glucosidase activity. The other three groups showed the most intense enzyme activities: the first group in which some strains of *T. pretoriensis*, *P. kudriavzevii*, and *S. cerevisiae* clustered more closely, showed high and moderate laccase activity, while a second group formed by some strains of *S. cerevisiae*, *H. burtonii*, *W. anomalus*, and *P. kudriavzevii* presented moderate and strong β-glucosidase activity, and the third group clustered the strains with intense laccase activity.

Our results show that the pattern of enzyme activities differentiates biotypes that include different yeast species. Moreover, no strain showed two strong enzyme activities at the same time, although cocoa beans might favour a high level of a specific enzyme activity by a single strain. Our results confirm that the yeasts here isolated were able to use specific substrates most commonly available in fermented and dry cocoa beans. For this reason, these strains can be considered potential starter cultures, due to the fact that they are well adapted to the ecological, environmental and processing conditions, and therefore they could be able to grow rapidly and dominate in the product.

### 3.4. Yeast Thermotolerance Is Modulated by Polyphenols Content

During cocoa fermentation and drying, one of the main abiotic stress factors influencing yeast growth and development is heat stress. The environmental temperature during Colombian beans fermentation varied between 21 and 33 °C. However, during fermentation, the temperature inside the fermenting pulp mass increased, reaching a maximum value of 46.5 °C (after 48 h) and 33 °C (after 126 h). During drying, a temperature of about 55 °C was observed, which could damage yeast cells membranes, hindering the cell capability to overcome desiccation and persist in the cocoa habitat. In our study, we found 12 different species that survived after air-drying, and some of them were not reported before as thermotolerant yeasts. The screening of thermal resistance in yeasts from cocoa had been performed by other researchers for some species, by using synthetic media without taking into consideration the conditions that yeasts deal with during fermentation, such as the presence of organic acids, pectin, ethanol, and polyphenols. Considering the lack of knowledge on the impact of these compounds on yeast thermotolerance, in this part of our study we compared directly the growth of the 42 selected yeasts strains that showed good enzyme activities during the screening, at 37 °C and 46 °C in YPD and CPSM.

**Figure 3:**
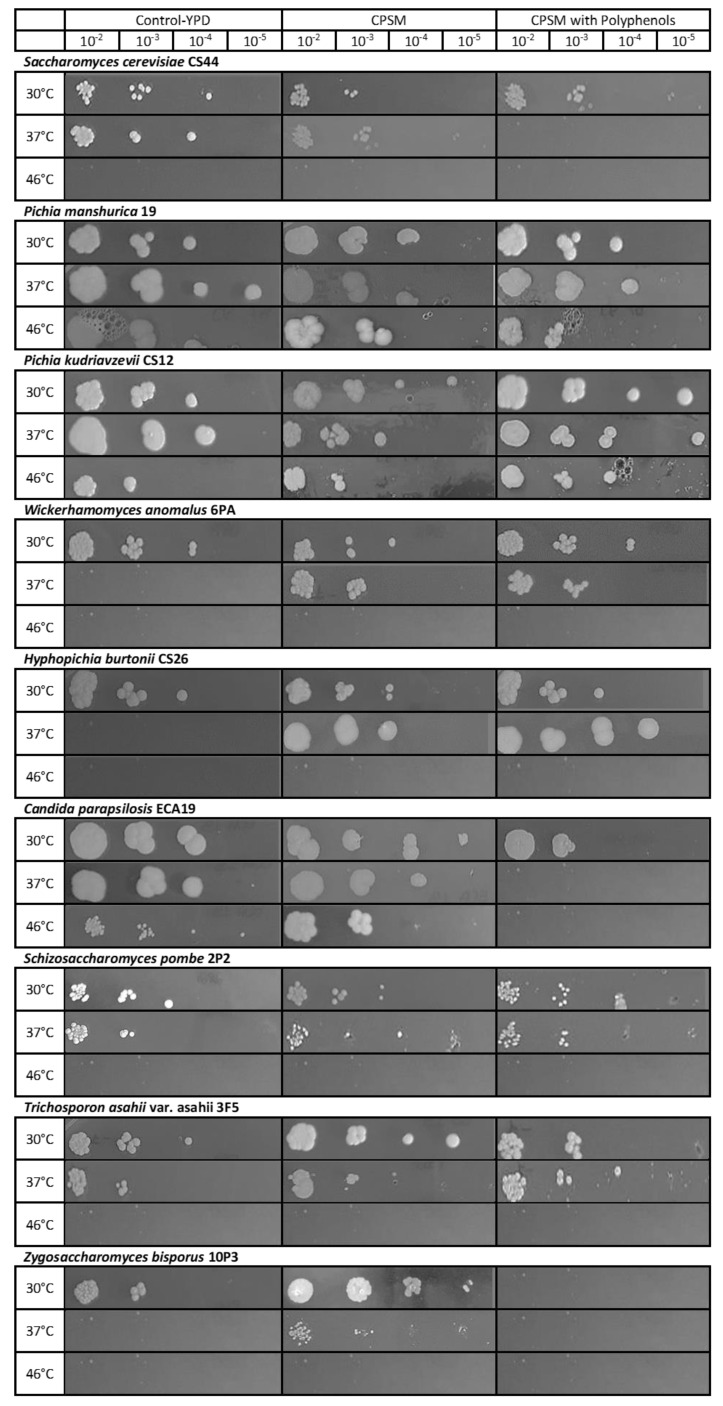
Effects of the medium composition on the thermotolerance of the different yeast species isolated from fermented and dry cocoa beans. CPSM: cocoa pulp simulation medium.

In general, considerable differences were found among the strains for thermotolerance at high temperatures, as growth was strain- and medium- dependent. In the experiments performed in YPD, not all the species were able to grow at 37 °C. The strains growing at this temperature belonged to the species *S. cerevisiae* (7/7), *P. manshurica* (3/3), *P. kudriavzevii* (7/7), *C. parapsilosis* (6/6), *T. asahii* var. *asahii* (3/3), and *H. burtonii* (1/7), although at 46 °C the number of strains able to grow and form colonies was reduced. As shown in Figure 3, only the growth of *P. manshurica* (2/3), *P, kudriavzevii* (5/7), and *C. parapsilosis* (1/6) was observed. Some earlier studies were addressed to the characterization of yeasts by identifying thermotolerant strains belonging to *P. kudriavzevii* and *S. cerevisiae* from fermented cocoa beans [28,33], and in industrial processes to improve the production of ethanol from sugar cane [46,47,48]. The thermotolerance of *P. manshurica* and *C. parapsilosis* isolated from fermented cocoa beans has never been reported before but a previous study found this characteristic in strains from soil samples collected from the Mekong Delta, in Vietnam [49], and from sugar cane from Brazil [48]. In addition, the thermotolerance of *C. parapsilosis* isolated from soil has been previously documented by El-Gamal et al., [50].

Although heat has its own detrimental effect on yeasts, the presence of other factors, including medium composition, age of the cells, aerobic, or anaerobic growth [51], may influence their thermal resistance. In particular, sugars as well as low *a_w_* values, are known to act as protectants against high temperature stresses [52]. Very recently, Peláez-Soto et al., [53] suggested that polyphenols from cocoa powder, and in particular flavonoids, were able to exert antioxidant activity in *S. cerevisiae*. On the contrary, Todorovic et al. [54] reported the efficacy of cocoa polyphenols to reduce the growth of *Candida albicans*. These evidences led us to think that polyphenols could greatly contribute to the growth response of the yeasts in CPSM, and for this reason we tested the ability to grow at 37 and 46 °C in CPSM with and without cocoa polyphenols. Our results indicate that CPSM modulated the heat stress response in different ways, and that polyphenols could effectively influence yeast thermoresistance. As observed, the yeast strains could be classified into two groups: (i) those with promoted growth, as in the case of *P. kudriavzevii* ECA 33 and CS12 at 46 °C, as well as the growth at 37 °C of *H. burtonii* CS26, CS29, CS21, CS 32, and *W. anomalus* CS21, 6PA, and 6PB; (ii) those with reduced cell growth at 37 °C, as in the case of *S. cerevisiae*, CS16, CS34, CS39, CS44, and *C. parapsilopsis* ECA34, ECA32, ECA31, ECA3. *P. manshurica* remained almost invariable. These results suggested a variable response of yeasts to cocoa polyphenols. Figure 3 shows a representative strain of each of the tested species.

Some studies proposed that the mechanisms of activity of polyphenols can go beyond their antioxidant activities, with possible modulatory effects on cell signalling pathways. For example, polyphenols were able to induce several genes to redirect carbohydrate metabolism towards the production of trehalose, whose levels increased 4-fold after exposure to quercetin. Moreover, polyphenols activity is related with cell integrity, as it shows a protective effect against oxidative stress [55]. Very recently, Peláez-Soto et al. [53] suggested that the protective effect of the cocoa polyphenols is mainly exerted through the regulation of: (a) amino acids metabolism by modulating the production of molecules with known antioxidant roles; (b) stress-responsive protein Yhb1; (c) protein Prb1, which can act by clipping Histone H3 N-terminal tails that are related to cellular resistance to DNA damaging agents. Our results showed that some low-thermotolerant species are more robust when tested in CPSM than in YPD, probably the high content of sugars and the presence of cocoa polyphenols in the medium could have contributed to this effect. From our results, it was not possible to explain the opposite effects of polyphenols in some of the yeasts here studied. For this reason, further research will be addressed to investigate the effects of the single compounds on these yeasts during thermal treatment.

Another interesting characteristic that we observed in some species was the change in colony morphology as a function of temperature or/and CPSM, which was very evident in *P. kudriavzevii* and *H. burtonii*. In this regard, Granek and Magwene [56], underlined that limitation of one or more key nutrients can trigger a variety of developmental responses in yeasts.

### 3.5. Differences in Trehalose Accumulation as a Response to Mild Heat Stress

In yeasts, several common mechanisms are important for the adaptation to the environment, and in particular the changes in composition of the membrane fatty acids, redox metabolism, production of proline and trehalose, production and transport of glycerol, and activity of various ion transporters [57,58]. Trehalose is a well-known storage carbohydrate for yeasts, contributing to the stabilization of biological membranes, proteins, and nucleic acids under stress conditions [59]. To obtain information on yeast biodiversity in the accumulation of molecules that could be useful to overcome the environmental heat stress, the selected strains were used to test their technological performance by evaluating the production of trehalose as a response to heat shock treatment at 46 °C in CPSM for 2 h.

**Figure 4:**
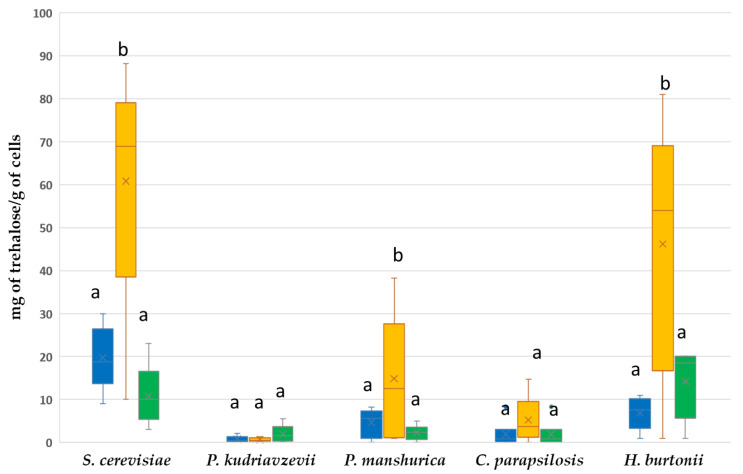
Trehalose content in yeasts isolated from Colombian fermented and dry cocoa beans exposed to heat shock treatment at 46 °C in CPSM liquid medium for 2 h. Blue: untreated control, Yellow: yeasts treated in CPSM; Green: yeasts treated in YPD. Values not sharing a letter in common differ significantly (*p* < 0.05 from each other) as determined by Duncan’s multiple range test.

Our results evidenced that in untreated cells a considerable inter-specific variation in trehalose content was observed (data not shown), with values ranging between 0.11 ± 0.03 (*C. parapsilosis*) and 81.20 ± 4.67 mg trehalose g^−1^ cell (*S. cerevisiae* CS34). After the thermal stress, the different strains accumulated trehalose in different quantities, with *P. manshurica* as the best accumulator (8 fold), followed by *S. cerevisiae* (5.9 fold), *H. burtonii* (5.4 fold) and *C. parapsilosis* (2.5 fold); on the contrary, *P. kudriavzevii* showed the lowest amounts of trehalose accumulation (0.75 fold) (Figure 4). Our data suggest that trehalose accumulation could help only some strains and species to withstand adverse heat stress. In fact, high trehalose accumulation could make the yeast cells resistant to multiple stresses, such as ethanol, heat, and freezing, although the importance of this accumulation before and after stress induction varied, depending on the type of stress [59]. Whilst high concentration of trehalose can stabilize proteins and membranes, enhancing cell thermotolerance [60], trehalose alone cannot explain the difference in thermotolerance among the cocoa yeasts here studied. It is well known that the transient and sub-lethal temperatures involve the induction of the synthesis of specific highly conserved stress proteins (Hsps), and it was proved that some of these proteins act together synergistically with trehalose to confer thermoprotection to *S. cerevisiae* [61].

Our findings are in line with other studies that demonstrated global transcriptomic changes in the yeast metabolism after thermal shock. For example, Mensonides et al. [62] found that the response of *S. cerevisiae* to a temperature shift from 28 to 41 °C led to a compromised cell growth and to an induction, in the first hour, of genes involved in energy metabolism and trehalose metabolism. In addition, the genes encoding for molecular chaperones were most significantly induced, while the genes encoding for components of translation and transcription machinery were provisionally down-regulated. On the other hand, *P. kudriavzevii*, in response to heat stress at 42 °C, down-regulated the expression of the genes involved in the trehalose metabolism and up-regulated the genes encoding for Hsp (ssq1 and hsp90), alcohol dehydrogenases (adh1, adh2, adh3, and adh4), and glyceraldehyde-3-phosphate dehydrogenase (tdh2). This adaptation may explain, in part, the survival of these yeasts at the end of drying of the cocoa-fermented mass. Great part of the literature regarding the mechanisms of thermoresistance in yeasts is prevalently focused on *S. cerevisiae* and *P. kudriavzevii*, while there is a lack of data regarding the other species here reported. In addition, the increased thermotolerance of some species such as *P. manshurica* and *C. parapsilopsis* was related to the presence of polyphenols in the medium, suggesting their fundamental role in shaping the yeast community composition of the cocoa beans during fermentation and drying.

## 4. Conclusions

The results of the present study indicate that *P. kudriavzevii*, followed by *S. cerevisiae* and *P. manshurica*, were well-adapted yeasts found in the samples of cocoa dry beans of the Colombian *Criollo* variety. In addition, we found that the studied yeasts differed in their enzyme activities, and this diversity could possibly affect cocoa beans fermentation. In fact, the differences in enzyme activities are considered as one of the major drivers of the important roles that yeasts carry out in cocoa fermentation, and are crucial for enhancing microbial nutrient availability and for promoting the growth of other microorganisms.

Our results suggest that the compounds, and in particular polyphenols, present in cocoa beans during drying could protect some yeasts against the high temperatures. However, it is important to consider that during the exposure of yeasts to drying, the temperature rises gradually because of the differences in thermal sensitivity of some components, which are not in contact with cocoa beans hot surfaces. This slow increase may act as a heat stress, which could lead to a heat resistance increase in a number of yeasts, increasing the general resilience of the cells to the conditions found in the cocoa fermented beans. This study is the first to report that yeast thermotolerance is modulated by polyphenols present in cocoa beans.

## Figures and Tables

**Table 1:** Total yeast counts, pH, aw and moisture determined in cocoa beans before and after drying.

Production Stage	Yeast Counts Log CFU/g	pH	*a*_w_	Moisture (%)
Before drying	6.59 ± 0.46 ^a^	5.3 ± 0.37 ^a^	0.98 ± 0.21 ^a^	42.6 ± 3.2 ^a^
After drying	2.73 ± 1.71 ^b^	5.1 ± 0.66 ^a^	0.52 ± 0.11 ^b^	3.4 ± 1.3 ^a^

Mean and standard deviation of 19 different samples analysed. Different lowercase letters indicate significant (*p* < 0.05) differences between the samples before and after sun drying.

**Table 2:** Identification of the yeast isolates determined by partial (D1/D2 loop) 26S rRNA encoding gene sequence analysis.

Strains	Closest Relative	Identity (%)	Accession Number
ECA30, ECA34, ECA32, ECA29, ECA31, ECA4, ECA3, ECA5, ECA19, ECA14.	*Candida parapsilosis*	99.83%	MH911007.1
CS27, CS31, CS26, CS24, CS20, CS29, CS32, CS49.	*Hyphopichia burtonii*	100%	CP024760.1
CS2, CS3, CS42, CS43, 7, ECA20, 3F3, 8P1, ECA6, 9P3, 1P8, CS1, CS4, CS19, CS18, CS17, CS6, CS 9, CS10, CS7, CS 8, CS 14, CS 15, 3F4, 10P2, 10P4, CS13, CS12, CS45, ECA33, ECA22, ECA23, ECA26, ECA38, ECA37, ECA39, O5?, ECA21, 19, ECA40 ECA41, ECA8, ECA7, ECA6, ECA10, ECA9, ECA11, ECA13, ECA12, ECA15, ECA16, ECA36, 8P8, 8P4, 8P9, 8P10.	*Pichia kudriavzevii*	100%	AY529502.1
3F ECA25, ECA24, 1, 14, ECA35, ECA18, ECA17, 2F1, ECA28, 16, 12, 19, CS28/3.	*Pichia manshurica*	100%	MK034750.1
6P2, 6F1.	*Torulaspora delbrueckii*	99.83%	MT255021.1
6F2, 6P3.	*Torulaspora pretoriensis*	99.43%	KY109883.1
P10, 8P5, 3F5.	*Trichosporon asahii* var. *asahii*	99.55%	KY109934.1
1P6, 2F3, 2F2, 1F1, 2F1, 1F2, 1P4, 9P2, CS39, CS44, 3, 5, 4, 8P7, 2, CS36, CS35, CS34, CS37, CS40, 10P4, CS1, CS23, ECA1, ECA2, CS25, 3F2, 8P2/1, 3P2, 3P1, 3P3, 15, 8P2, 8P6, CS41, 17, CS16, 8P5/1, CS11.	*Saccharomyces cerevisiae*	99.66%	JX141382.1
CS50, 8.	*Starmerella sorbosivorans*	99.55%	NG_060827.1
6PA, 6PB, 6P1/b, 9P1, 6PB/1, 10, CS2/1.	*Wickerhamomyces anomalus*	99.66%	MT255020.1
10P1, 10P3.	*Zygosaccharomyces bisporus*	99.49%	NG058447.1
CS48, CS47, CS46, 2P2.	*Schizosaccharomyces pombe*	99.84%	KY109602.1

**Table 3:** Frequency and abundance of yeast species isolated from *Criollo* Colombian cocoa samples, fermented for 6 days and sun dried for 3 to 6 days.

Yeast Species	Frequency ^a^	Abundance ^b^ (%)
*Candida parapsilosis*	5/19	6.6
*Hyphopichia burtonii*	2/19	5.18
*Pichia kudriavzevii*	15/19	40.00
*Pichia manshurica*	7/19	8.88
*Saccharomyces cerevisiae*	15/19	28.14
*Schizosaccharomyces pombe*	2/19	2.77
*Starmerella sorbosivorans*	2/19	1.48
*Torulaspora delbrueckii*	2/19	1.38
*Torulaspora pretoriensis*	1/19	1.48
*Trichosporon asahii* var. *asahii*	2/19	2.01
*Wickerhamomyces anomalus*	4/19	4.4
*Zygosaccharomyces bisporus*	2/19	1.48

^a^ number of samples in which the species was present. ^b^ number of isolates identified of each species out of the total number of isolates.

## Supplementary Materials

The following are available online at https://www.mdpi.com/2076-2607/8/7/1086/s1, Table S1: Description of study sites and diversity indexes of yeasts included in the present study.
