# Supplementary material for: Functional Biodiversity of Yeasts Isolated from Colombian Fermented and Dry Cocoa Beans
Source: Microorganisms. 2020 Jul 21;8(7):1086. doi: 10.3390/microorganisms8071086 (PMC7409280; doi:10.3390/microorganisms8071086)
Supplement: Supplementary file 1 [file microorganisms-08-01086-s001.pdf]

Table S1. Description of study sites and diversity indexes of yeasts included in the present study.

sample	Geographical coordinates		Type of drying	Drying Temperature (°C)		days of drying	a_w	Log CFU·g^{-1}	Shannon Index H'
	Latitude	Longitude		Day	Night				
1	03°10'55''N	76°27'37''W	floors	25-27	18-19	5	0.421	4.44 ± 0.38	2.67
2	4°07′12″N	76°13′17″W	Trays	28-30	17-18	4	0.501	3.46 ± 0.17	2.10
3	4°09′03″N	76°14′28″W	Trays	28-30	17-18	5	0.534	1.80 ± 0.25	2.59
4	4°07′07″N	76°13′13″W	Trays	25-27	18-19	3	0.550	1.00 ± 0.48	1.67
5	4°07′18″N	76°12′41″W	Trays	28-30	17-18	3	0.535	1.50 ± 0.33	2.43
6	03°11′44″N	76°19′44″W	Trays	29-30	18-19	5	0.650	5.63 ± 0.40	2.08
7	4°07′20″N	76°12′16″W	floors	28-30	17-18	6	0.321	1.50 ± 0.50	1.69
8	4°08′07″N	76°12′43″W	Trays	25-27	18-19	4	0.542	5.24 ± 0.81	2.24
9	4°08′24″N	76°13′11″W	Trays	25-27	18-19	4	0.623	3.12 ± 0.80	1.00
10	03°13′58″N	76°17′38″W	floors	29-30	18-19	6	0.449	2.55 ± 0.13	2.10
11	03°14′20″N	76°16′18″W	floors	28-33	17-20	6	0.422	1.50 ± 0.51	1.00
12	4°07′12″N	76°12′08″W	floors	28-30	17-18	4	0.440	1.69 ± 0.76	1.68
13	4°07′18″N	76°13′08″W	floors	28-30	17-18	3	0.450	0.00 ± 0.46	1.00
14	4°07′21″N	76°13′08″W	Trays	25-27	18-19	3	0.662	1.84 ± 0.50	1.00
15	03°14′30″N	76°16′01″W	Trays	29-30	18-19	5	0.806	5.47 ± 0.56	2.30
16	4°07′21″N	76°13′08″W	wooden	28-30	17-18	5	0.469	2.12 ± 0.14	1.64
17	1°43′27″N	78°48′42″W	Trays	20-25	11-15	4	0.560	1.47 ± 0.10	1.00
18	4°07′21″N	76°13′14″W	floors	28-30	17-18	4	0.459	1.59 ± 0.20	1.00
19	4°07′21″N	76°13′20″W	Trays	25-27	18-19	3	0.651	5.19 ± 0.28	1.50
